# Supplementary material for: Performance evaluation of the Ortho VITROS SARS-CoV-2 Spike-Specific Quantitative IgG test by comparison with the surrogate virus neutralizing antibody test and clinical assessment
Source: PLoS One. 2023 Jan 24;18(1):e0279779. doi: 10.1371/journal.pone.0279779 (PMC9873150; doi:10.1371/journal.pone.0279779)
Supplement: Attachment — Submitted filename: Response to Reviewers_Takahashi_R1.docx [file pone.0279779.s001.docx]

September 14, 2022

Dr. Etsuro Ito

Academic Editor

PLOS ONE

RE: Resubmission of the revised manuscript MS# PONE-D-22-15685 titled “Performance evaluation of the Ortho VITROS SARS-CoV-2 Spike-Specific Quantitative IgG test by comparison with neutralizing antibody and clinical assessment” by Takahashi M., et al.

Dear Dr. Etsuro Ito,

We appreciate the reviewers for their highly constructive comments for our manuscript, and we believe that we have been able to fully address the comments as detailed below.

Reply (Answer; A) to Reviewer’s comments and questions (Comment; C):

**COMMENTS by Reviewer #1:**

Takahashi M. et al. evaluated the performance of the Ortho VITROS SARS-CoV-2 Spike-Specific Quantitative IgG kit. This work would give the useful information to the readers, but it is necessary to improve this manuscript for publication in PLoS One.

**C1:** Line 229: Table 1 does not summarize the clinical background characteristics. Please add the table to show the clinical information.

**A1:** We apologize for our carelessness. We summarized the clinical background information in **Table 5**.

**C1:** Fig. 1: The power of antibody detection seems different between cPass sVNT and VITROS S-IgG. Why? Please explain the reason.

**A2:** Following the reviewer’s comments, we discussed the difference in the power of antibody detection between VITROS S-IgG and cPass sVNT. **(Table 2,　Line 371-383)**

**C3:** Fig. 2: Fig. 2 (A), (B), and (C) are redundant. Please avoid the repetition. (I think that all results shown in (A), (B), and (C) can be unified in (C).)

**A3:** Following the reviewer’s suggestion, we deleted Fig.2 (A) and (B) and merged them into **Figure 2.**

**C4:** Fig.3: Fig. 3 (A), (B), and (C) are also redundant. Please avoid the repetition.

**A4:** We removed Fig. 3 (A) and (B) and unified them in **Figure 3** as suggested by the reviewer.

**C5:** Please show and discuss the specificity and sensitivity of VITROS S-IgG and cPass sVNT. Authors should clearly state the advantages and disadvantages of VITROS S-IgG as compared with cPass sVNT.

**A5:** Clinical sensitivities of VITROS S-IgG and cPass sVNT were shown in **Tables 3 and 4.** The manufacturer's claimed sensitivity and specificity for his VITROS S-IgG and cPass sVNT were further shown in **Table 1.** The lack of original specificity data was stated as a study limitation. **(Line 413-414)**

We discussed the advantages and disadvantages of VITROS S-IgG compared to cPass sVNT. **(Line 421-427, 430-434).**

**C6:** I recommend adding the new table for easy understanding of the details of VITROS S-IgG and cPass sVNT (manufacturer, method, antigen, immunoglobulin class, unit etc.).

**A6:** Following the reviewer’s suggestion, we made **Table 1** describing the specifications of VITROS S-IgG and cPass sVNT.

**C7:** The amount of antibody can be affected by how long time has passed after vaccination. However, there was no information on the timing of sample collection from the vaccinees. Please show this point.

**A7:** We analyzed the changes of antibody titers over time after vaccination, and discussed the results **(L334-337, L401-411, Figure 6)**

**C8:** In addition, no information was shown when the patients in Group 3 were vaccinated and diagnosed with COVID-19. The timings of vaccination, sample collection, and infection should influence the antibody titer. Please clarify these points.

**A8:** Following the reviewer’s suggestion, we described the timings of vaccination, sample collection, and infection **(Line 318-317, Line 328).**

**COMMENTS by Reviewer #2:**

In this manuscript, Takahashi et al evaluated the performance of Ortho VITROS SARS-CoV-2 spike-specific quantitative IgG (VITROS S-IgG) assay, in comparison with GenScript cPass SARS-CoV-2 Neutralization Antibody Detection Kit (cPass sVNT assay). They described that VITROS S-IgG showed good correlation with the cPass sVNT assay. They concluded that VITROS S-IgG is useful as a diagnostic tool and can be utilized for assessing immune response to vaccination and herd immunity.

This is the first report to show the correlation of VITROS S-IgG with NT antibodies, but there are several points which have to be improved for publication in PLoS One.

Major points

**C1:** Page 7, lines 108 to 112.

The authors should describe the details of the patients’ characteristics of Group S and Group M, which are explained in the COVID-19 clinical management Living Guideline from WHO.

**A1:** We apologize for the insufficient explanation. A detailed description of the groupings according to the WHO classification has been provided. **(Line 110-123)**

**C2:** P2age 6, lines 90 to 94.

cPass sVNT assay is a surrogate test for neutralizing antibody using pseudovirus as the authors mentioned in the abstract and the main text; the comparison shown in this manuscript was not performed using cell-culture test which is the gold standard method for virus-neutralization. The authors should describe it as a limitation of this study in the discussion.

**A2:** We agree with the reviewer’s comment. We discussed that “further research is needed to determine whether sVNT detected neutralizing antibody levels are directly related to protection against infection”. **(Line 381-383)** And noted that we did not perform the 'gold standard' PRNT, as a limitation of the study. **(Line 418-420)**

**C3:** Figure 1 and Table 1

There are few description regarding Table 1 in the main text. Assuming that Figure 1 and Table 1 show the same content, the correlation between VITROS S-IgG and cPass sVNT assay is not excellent; The agreement of result calculated from Table 1 is 85%. The authors should explain it in the discussion.

**A3:** Thank you for the valuable comments. We corrected **Table 2** and the description of the main text as follows; “The concordance between the qualitative results of the VITROS S-IgG and the cPass SARS-CoV-2 neutralization test in SARS CoV-2 positive patients are shown in **Table 2**. The positive percent agreement of the VITROS S-IgG with cPass sVNT was 85.0 % (198/233), and the negative percent agreement was 84.1 % (37/44). The overall percent agreement of the VITROS S-IgG with cPass sVNT was 84.8% (235/277). Among the VITROS S-IgG positive samples, 96.6 % (198 / 205) showed positive for cPass sVNT. However, we observed that 48.7% of the VITROS S-IgG negative samples were cPass sVNT positive (35/72).” **(Line 202-209)** We also discussed about these findings in the discussion section. **(Line 371-383)**

Minor points

**C4:** Page 8, lines 116 to 118.

The approvable number from Juntendo IRB is required.

**A4:** We stated the IRB number. **(Line 128)**

**C5:** Page 14, line 229.

Table 1 shows “Agreement between Vitros S-IgG and cPass”, but not “summarizes the clinical background characteristics”. The authors should reconcile these expression. In addition, the order of items in the table 1 should be corrected; VITROS S-IgG should be shown in the left column, rather than in the upper right in the table. The explanation for “(%)” is needed, because it is hard to understand which ratio is shown as “(%)”. “Positive” should be shown preferentially upward than “Negative”.

**A5:** We apologize for our carelessness. Clinical background characteristics of the patients was summarized in **Table 5**. Agreement between the VITROS S-IgG and the cPass sVNT was shown in **Table 2.**
