# Supplementary material for: Performance evaluation of the Ortho VITROS SARS-CoV-2 Spike-Specific Quantitative IgG test by comparison with the surrogate virus neutralizing antibody test and clinical assessment
Source: PLoS One. 2023 Jan 24;18(1):e0279779. doi: 10.1371/journal.pone.0279779 (PMC9873150; doi:10.1371/journal.pone.0279779)
Supplement: Attachment — Submitted filename: Response to Reviewers_Takahashi_R2_1130.docx [file pone.0279779.s002.docx]

December 1, 2022

Dr. Etsuro Ito

Academic Editor

PLOS ONE

RE: Resubmission of the revised manuscript MS# PONE-D-22-15685R1 titled “Performance evaluation of the Ortho VITROS SARS-CoV-2 Spike-Specific Quantitative IgG test by comparison with neutralizing antibody and clinical assessment” by Takahashi M., et al.

Dear Dr. Etsuro Ito,

We wish to express our appreciation to the reviewers for their insightful comments on our paper. The comments have helped us to improve the paper.

Reply (Answer; A) to Reviewer’s comments and questions (Comment; C):

**COMMENTS by Reviewer #1:**

 I feel that the quality of the revised manuscript has been much improved, but this version still has some points to be changed for considering publication in PLoS One.

**C1**: I would like to emphasize that cPass sVNT assay does not show NT titers but the inhibition values of ACE2-RBD interaction as the surrogate based on ELISA. The use of the word "neutralizing antibody" or "NT" would cause the readers to misunderstand. I recommend using other words like "surrogate neutralizing antibody" or "sVNT" in the whole manuscript. Please consider the revision of the title, in particular.

**A1: Following the reviewer's suggestion, we used the term "surrogate virus neutralizing (sVN) antibody" or "surrogate virus neutralization test (sVNT) value" for the antibody measured by the cPass sVNT assay throughout the manuscript. We also revised the title.**

**C2**: The authors concluded that VITROS S-IgG shows good correlation with the cPass sVNT assay. However, I feel that the interpretation of the results by the authors was not reasonable. Table 2 demonstrates that the performance of VITROS S-IgG was quite different from that of cPass sVNT. Table 3 and Table 4 also show that cPass sVNT had the higher clinical sensitivity than the VITROS S-IgG (for example, cPass sVNT assay showed 4 positives in 7 samples, but VITROS S-IgG assay did 0 positive in 20 samples in mild cases). Fig.1 to 4 clearly exhibited that VITROS S-IgG had the lower sensitivity than cPass sVNT.

**A2: We agree with the reviewer’s comment, and have revised the abstract and discussion. (Line 61-65, Line 377-384)**

**C3**: Previously, I requested the authors to explain the reason why the power of antibody detection was higher in cPass sVNT than VITROS S-IgG. However, the explanation in the revised manuscript was still insufficient.

**A3: We revised the discussion section (Line 377-384).**

**COMMENTS by Reviewer #3:**

 The authors compared the clinical performance of VITRO S-IgG and the NT antibody levels (sVNT assay) using 332 serum samples collected from 188 individuals. The samples were grouped into Group S (severe or critical) and Group M (mild or moderate) with 113 samples obtained from healthcare workers who had received two doses of BNT162b2 vaccine.
The manuscript is well written, and the methodology of the research and its statistical analysis is in a sound manner. In addition, the authors adequately have followed the suggestions of the reviewers and revised their manuscript appropriately. Although these may be trivial things, the manuscript seems to contain some erroneous grammatical usages of English. They should be amended before the manuscript reaches the decision of acceptance for its publication in PLoS One. It is strongly recommended that the manuscript should undergo English editing services. Furthermore, there is a minor point which should be considered for the authors to revise, as below.

**C1**: A minor point: in line 310(R1), table 5 is not informative and should be omitted. This table only presents the number of the patients accompanied by their disease severity, group, sex, and past medical history, all of which are not relevant to the substance of this research. This information can be summarized and simply included in the main text.

**A1: Following the reviewer’s suggestion, we deleted table 5 and added this information in the main text. (Line 292-295)**

**COMMENTS by Reviewer #4:**

The manuscript is well written but a few changes are recommended.

**C1**: In the abstract, this sentence is unclear and (N-specific) is not defined.
"In regard to the COVID-19 humoral immune response after the second dose of the BNT162b2 vaccination, similar levels of VITROS S-IgG and cPass sVNT were observed with high titers in N-specific seropositive individuals in both VITROS S-IgG and cPass sVNT."

**A1: Following the reviewer’s suggestion, we revised the abstract. (Line 53-58)**

**C2**: In the discussion (line 391) the is a paragraph about complement, T cells. This manuscript looked at neither complement nor T cells and the paragraph seems speculative and not based on the current data.

**A2: We agree with the reviewer’s comment and deleted the sentence.**

**C3**: Table 4 is cutoff on right side. There are 2 blank pages.

**A3: We apologize for our carelessness. We revised the pages.**
